# Dose-response association between physical activity and clustering of modifiable cardiovascular risk factors among 26,093 Chinese adults

**DOI:** 10.1186/s12872-020-01627-6

**Published:** 2020-07-25

**Authors:** Rui Shi, Yamei Cai, Rui Qin, Yang Yan, Dahai Yu

**Affiliations:** 1 Department of Cardiovascular Medicine, The First Affiliated Hospital of Xi’an Jiaotong University, Xi’an, 713300 China; 2 Jiangsu Province Hospital on Integration of Chinese and Western Medicine, Nanjing, 210028 China; 3 Department of Cardiovascular surgery, The First Affiliated Hospital of Xi’an Jiaotong University, Xi’an, 713300 China; 4 Primary Care Centre Versus Arthritis, School of Primary, Community and Social Care, Keele University, Keele, ST5 5BG UK

**Keywords:** Physical activity, Cardiovascular diseases, Cardiovascular risk factor

## Abstract

**Background:**

There is uncertain evidence in the dose-response association between overall physical activity levels and clustering of cardiovascular diseases modifiable risk factors (CVDMRF) in Chinese adults. This study examined the hypothesis whether inverse dose-response association between overall physical activity levels and clustering of CVDMRF in Chinese adults exist.

**Methods:**

Twenty-six thousand ninety-three Chinese adult participants were recruited by two independent surveys in Nanjing and Hefei during 2011 to 2013, from random selected households provided smoking, glucose, lipids, anthropometric, and blood pressure measurements. Logistic regression model was applied to examine the dose-response association between overall physical activity (measured by metabolic equivalent task (MET)- minutes per week) and having ≥1, ≥2, and ≥ 3 CVDMRF (dyslipidemia, hypertension, diabetes, cigarette smoking, and overweight).

**Results:**

An inverse linear dose-response relationship between physical activity and clustering of CVDMRF was identified, as increased physical activity levels are associated with lower odds of having clustering of CVDMRF. The adjusted odds ratio (95% confidence interval) of having ≥1, ≥2, and ≥ 3 CVRF for moderate physical activity group and high physical activity group was 0.88 (0.79 to 0.98) and 0.88 (0.79 to 0.99), 0.85 (0.78 to 0.92) and 0.85 (0.78 to 0.92), 0.84 (0.76 to 0.91) and 0.81 (0.74 to 0.89), respectively, with low physical activity as reference group.

**Conclusions:**

Among Chinese adults, physical activity level inversely associates with clustering of CVDMRF, especially in those aged 35–54 years. Health promotion including improve physical activity should be advocated. The potential role of physical activity in the clustering of CVDMRF warrants further validation.

## Background

Cardiovascular diseases (CVD) as the leading death cause has affected 290 million Chinese people with CVD and caused 3.5 million annual death [1]. It is also projected that annual cardiovascular events will increase by more than 50% by 2030 in China based on the current the aging population and the growth of population [2]. Different from the developed countries in which the prevalence of some CVD modifiable risk factors has decreased, the prevalence of CVD modifiable risk factors has increased in developing countries. In particular, major modifiable risk factors: current smoking, overweight, diabetes, hypertension and dyslipdaemia have been found very common in Chinese adults [3]. For example, it has been estimated that 80.5, 45.9, and 17.2% of Chinese adults had ≥1, ≥2, and ≥ 3 above CVD modifiable risk factors [3].

Emerging evidence suggests physical activity may be relevant to circulating metabolites, including lipoproteins, lipids, and glucose, which have been associated with risk of CVD [4, 5]. However, few studies have addressed the association between physical activity and clustering of CVD modifiable risk factors, particularly in Chinese population. The goal of this study was to quantify the dose-response association between physical activity level and having a clustering of 1 or more, 2 or more, and 3 or more of CVD modifiable risk factors: dyslipidemia, hypertension, diabetes, current smoking, and overweight in Chinese adults.

## Methods

### Data setting

Two independent surveys were implemented in two Chinese places: Nanjing and Hefei [6]. The Nanjing Community Cardiovascular Risk Survey was processed by random cluster sampling [7], between 2011 and 2013, among adults resident in 6 communities of Nanjing, the capital city of Jiangsu Province in China. In every Nanjing community, one township or street district was chosen at random. Six thousand four hundred forty-five households in the chosen town or street were incorporated with only one adult aged 20 years and over sampled from every household, without replacement. Five thousand eight hundred twenty-four participants completed their clinical examination and survey (response rate: 90%). The Hefei Community Cardiovascular Risk Survey was also implemented by a random cluster sampling [6], between 2012 and 2013 among the adults residents in 10 rural areas of Hefei, the capital city of Anhui Province in China. In every rural area of Hefei, one township was randomly chosen. Twenty-two thousand thirty-two households in the chosen rural town were all incorporated with only one adult aged 20 years and over sampled from every household, without replacement. Twenty thousand two hundred sixty-nine participants completed their clinical examination and survey (response rate: 92%). Ethics approval of this study was acquired from the Institutional Review Board of Jiangsu Province Hospital on Integration of Chinese and Western Medicine (approval number 11–006). Signed, Informed consent was obtained from all study participants.

### Clinical measurements

Both in Nanjing and Hefei surveys, research questionnaires were completed by trained research staff through face-to-face interviews. Question items covered age, gender, race, smoking status, and known diabetes. Participants who reported having smoked ≥100 cigarettes during their lifetime were classified as current smokers if they answered affirmatively to the question, “Do you smoke cigarettes now?” [3].

Both in Nanjing and Hefei surveys, body measurements and blood pressure measures were taken three times on the same day by a standardized methodology in the local clinical center and the mean of the two closest recordings was recorded as the final measurement. Body measurements including weight, height and waist circumference were measured by using a vertical weight scale and metric scale. Study participants’ weight were measured to the nearest 10th of a kilogram with wearing light indoor clothing without shoes. Study participants’ height was measured to the nearest 10th of a centimeter without wearing shoes. Both in Nanjing and Hefei surveys, above measurements were made by trained observers who were all asked to attend a training session to learn the standardized protocol of measurement techniques [8]. Study participants’ body mass index was calculated by their body weight (unit: kg) divided by the square of their height (unit: meter). Study participants with BMI ≥25 kg/m^2^ was defined as overweight [9].

Blood samples were collected for all participants after an overnight fasting for at least 10 h by trained nurse. The fasting time was verified prior to collecting the blood specimen. Participants who had not fasted for at least 10 h did not have their blood drawn.

Plasma samples for measuring glucose were collected using vacuum blood collection tubes containing anticoagulant sodium fluoride and the serum samples for measuring lipid profile and creatinine were collected using vacuum blood collection tubes not containing sodium fluoride. Fasting blood specimens collected in Nanjing were processed at the examination center (Nanjing) for urban population and fasting blood specimens collected in Hefei rural population were shipped by air to Nanjing examination centre. All specimens were stored at − 70 °C until laboratory assays were performed. Plasma glucose, serum creatinine, and serum lipid levels were measured by automated analyser (Olympus AU600 autoanalyser (Olympus Optical, Tokyo, Japan)). Participants who currently accepting anti-hypertensive treatment or having a 140 mmHg and over of a mean systolic blood pressure (SBP) and/or a 90 mmHg and over of mean diastolic blood pressure (DBP) were defined as having hypertension [10, 11]. Dyslipidemia was defined as currently accepting lipid-lowering therapy or having ≥1 of the higher lipid profile measurements as:

total cholesterol ≥5.2 mmol/L, triglycerides ≥1.7 mmol/L, HDL cholesterol < 1.0 mmol/L, or LDL cholesterol ≥3.4 mmol/L [12]. Participants currently accepting anti-diabetic therapies (insulin or oral hypoglycemic agents) or having a high measurement of fasting glucose (fasting plasma glucose level ≥ 7.0 mmol/L) was defined as having diabetes [13].

### Physical activity measurements

International Physical Activity Questionnaire was used to collect physical activity information on household, transport, and job, as well as those on leisure-time, sports and recreation. Information about ≥10 min specific activities by the intensity, daily duration, and total weekdays during the prior week was acquired from participants [14]. Metabolic equivalent task (MET)- minutes every week (Met Score) were generated from the raw data. Met Score was also grouped into 3 levels: low physical activity group (who having < 600 MET-min/week), moderate physical activity group (who having 600–3000 MET-min/week) and high physical activity group (who having ≥3000 MET-min/week) [15]. The MET Score was transformed to its square root due to the non-normality of distribution.

### Statistical analysis

The median and interquartile range was presented for continuous variables. Transformed Met Score (square root of Met Score) were compared by t test. Categorical variables were presented as count and percentages, and their difference across categories were examined by Chi-square test. Multivariable Logistic regression model was applied to assess the association between the transformed Met Score and odds of having ≥1, ≥2, and ≥ 3 major CVD modifiable risk factors by i) quantifying the odds ratio (OR) by increase of 1 standard deviation (SD) of transformed Met Score; ii) quantifying the OR by comparing the physical activity moderate and high level with the low level. Dose-response relationship between the transformed Met Score and odds of having ≥1, ≥2, and ≥ 3 major CVD modifiable risk factors was also examined by natural cubic spline model.

All statistical analyses were processed using STATA MP 15.0. All *P*-values were estimated by two-tailed tests and a *P*-value < 0.05 was taken as significance level.

## Results

The characteristics of study participants in Nanjing survey sample, Hefei survey sample and the pooled survey samples are presented in Table 1. The prevalence of current smoking, overweight, dyslipidemia, hypertension, and diabetes was 24.0, 44.2, 50.0, 44.0 and 8.0% in the pooled survey sample, respectively. The prevalence of each CVRF above was 28.0, 40.2, 44.4, 38.8, and 8.1% in Nanjing survey sample and 23.3, 45.3, 51.6, 45.5 and 9.0% in Hefei sample, respectively. The prevalence of having ≥1, ≥2 and ≥ 3 CVD modifiable risk factors was 83.6, 54.9 and 25.7% in the pooled survey sample, respectively. The prevalence of having ≥1, ≥2 and ≥ 3 CVD modifiable risk factors was higher in Hefei survey sample comparing with Nanjing sample, as 84.5, 56.4, and 26.8% in Hefei survey sample and 80.6, 50.0 and 21.9% in Nanjing survey sample, respectively.

**Table 1:** Characteristics of study participants

Characteristics	Survey-1 (Nanjing)	Survey-2 (Hefei)	Pooled data
Participants, n	5824	20,269	26,093
Age, years	52.0 (43.0 to 59.0)	51.0 (43.0 to 58.0)	51.0 (43.0 to 58.0)
Women, n (%)	3278 (56.3)	11,905 (58.7)	15,183 (58.2)
Current smoking, n (%)	1571 (28.0)	4687 (23.3)	6258 (24.0)
Hypertension, n (%)	2259 (38.8)	9228 (45.5)	11,487 (44.0)
Type 2 diabetes, n (%)	472 (8.1)	1832 (9.0)	2304 (8.8)
Body mass index, kg/m^2^	23.6 (21.4 to 26.1)	24.1 (21.8 to 26.5)	24.0 (22.1 to 26.8)
Waist circumference, cm	80.0 (73.3 to 87.0)	81.6 (74.3 to 88.7)	81.3 (74.0 to 88.4)
Systolic blood pressure, mmHg	128 (116 to 143)	132 (120 to 148)	131 (119 to 147)
Diastolic blood pressure, mmHg	80.5 (73.5 to 88.5)	82.5 (75.0 to 90.5)	82.0 (75.0 to 90.0)
Fasting glucose, mmol/L	5.4 (4.9 to 5.9)	5.3 (4.8 to 5.2)	5.3 (4.8 to 5.8)
Triglyceride, mmol/L	1.2 (0.8 to 1.7)	1.3 (0.9 to 1.8)	1.2 (0.9 to 1.8)
Total cholesterol, mmol/L	4.4 (3.9 to 4.9)	4.6 (4.0 to 5.2)	4.5 (4.0 to 5.1)
High density lipoprotein cholesterol, mmol/L	1.3 (1.1 to 1.5)	1.3 (1.1 to 1.5)	1.3 (1.1 to 1.5)
Low density lipoprotein cholesterol, mmol/L	2.4 (2.0 to 2.9)	2.6 (2.2 to 3.1)	2.5 (2.1 to 3.0)
Dyslipidaemia, n (%)	2583 (44.4)	10,463 (51.6)	13,046 (50.0)
Overweight, n (%)	2341 (40.2)	9187 (45.3)	11,528 (44.2)
Having ≥1 CVD modifiable risk factor, n (%)	4692 (80.6)	17,118 (84.5)	21,810 (83.6)
Having ≥2 CVD modifiable risk factors, n (%)	2914 (50.0)	11,421 (56.4)	14,335 (54.9)
Having ≥3 CVD modifiable risk factors, n (%)	1275 (21.9)	5422 (26.8)	6697 (25.7)

Continuous variables were presented as median (interquartile range); categorical variables were presented as number (percentage)

The distribution of physical activity levels (transformed Met Score) was presented in Table 2 by the status of clustering of CVD modifiable risk factors. Participants with clustering of CVD modifiable risk factors was more likely to have lower physical activity comparing with those without clustering of CVD modifiable risk factors, as the median (IQR) of transformed Met Score was 48.9 (33.8 to 70.2), 47.8 (33.6 to 70.7) and 46.2 (32.9 to 69.1) among those with ≥1, ≥2 and ≥ 3 CVRF; 50.0 (34.1 to 71.9), 50.2 (34.1 to 72.0), and 50.1 (34.2 to 72.0) among those without ≥1, ≥2 and ≥ 3 CVD modifiable risk factors, respectively. Similarly, significantly lower physical activity was found among participants with clustering of CVD modifiable risk factors comparing with those without clustering of CVD modifiable risk factors in each survey sample and by sex. In the age-stratified analysis, the significant difference was found in age group 35–44 years and 45–54 years.

**Table 2:** Distribution of physical activity levels by cardiovascular disease risk factors clustering status among Chinese adults

	Having ≥1 CVD modifiable risk factor		*P*	Having ≥2 CVD modifiable risk factors		*P*	Having ≥3 CVD modifiable risk factors		*P*
Met Score, median (Inter-quartile range)	Yes	No		Yes	No		Yes	No	
Overall	48.9 (33.8 to 70.2)	50.0 (34.1 to 71.9)	0.017	47.8 (33.6 to 70.7)	50.2 (34.1 to 72.0)	0.003	46.2 (32.9 to 69.1)	50.1 (34.2 to 72.0)	< 0.001
Survey site									
Nanjing	44.2 (29.2 to 68.0)	47.1 (31.0 to 69.3)	0.028	43.5 (28.5 to 67.1)	45.7 (30.5 to 69.2)	0.013	43.0 (27.6 to 64.6)	45.4 (30.2 to 69.3)	0.003
Hefei	49.6 (35.3 to 71.9)	51.3 (35.5 to 72.2)	0.043	50.2 (35.4 to 71.3)	51.2 (35.5 to 72.7)	0.002	50.0 (34.5 to 69.8)	51.4 (35.5 to 72.7)	< 0.001
Sex									
Male	48.3 (32.5 to 72.7)	51.4 (33.5 to 76.1)	0.010	47.0 (32.3 to 71.9)	50.0 (33.2 to 74.9)	0.002	46.1 (31.5 to 70.2)	50.0 (33.3 to 74.4)	0.002
Female	49.6 (34.2 to 68.2)	50.0 (43.6 to 71.0)	0.183	48.3 (34.9 to 69.2)	51.4 (34.6 to 71.0)	0.012	48.1 (34.6 to 68.2)	50.6 (34.7 to 71.0)	0.003
Age group									
< 35 years	46.6 (30.5 to 62.0)	47.3 (33.4 to 68.9)	0.098	44.4 (25.8 to 62.2)	47.3 (35.5 to 86.0)	0.072	48.3 (17.8 to 63.4)	47.3 (32.9 to 93.1)	0.098
35–44 years	48.8 (32.4 to 73.2)	49.3 (33.5 to 75.3)	0.047	47.9 (31.3 to 73.2)	49.3 (33.5 to 75.1)	0.016	46.7 (30.0 to 73.6)	49.6 (33.2 to 74.8)	0.037
45–54 years	48.5 (34.5 to 72.7)	51.0 (35.5 to 73.5)	0.036	47.3 (34.8 to 72.9)	50.8 (34.5 to 73.4)	0.026	49.4 (33.4 to 71.6)	51.4 (35.1 to 73.9)	0.021
55–64 years	49.1 (33.3 to 67.9)	50.0 (34.4 to 68.7)	0.056	50.0 (32.4 to 67.3)	50.3 (34.3 to 69.1)	0.072	49.1 (32.2 to 67.5)	50.2 (34.6 to 69.4)	0.063
65–74 years	49.5 (34.0 to 65.1)	50.0 (32.9 to 67.7)	0.062	48.9 (33.9 to 65.1)	49.8 (34.0 to 65.8)	0.083	49.1 (33.7 to 65.4)	50.4 (35.3 to 64.9)	0.078
≥ 75 years	49.7 (29.4 to 68.1)	50.1 (43.7 to 89.6)	0.056	48.9 (27.9 to 68.1)	49.8 (36.3 to 78.4)	0.076	46.2 (24.5 to 53.9)	49.2 (39.5 to 79.2)	0.061

**Fig. 1:**
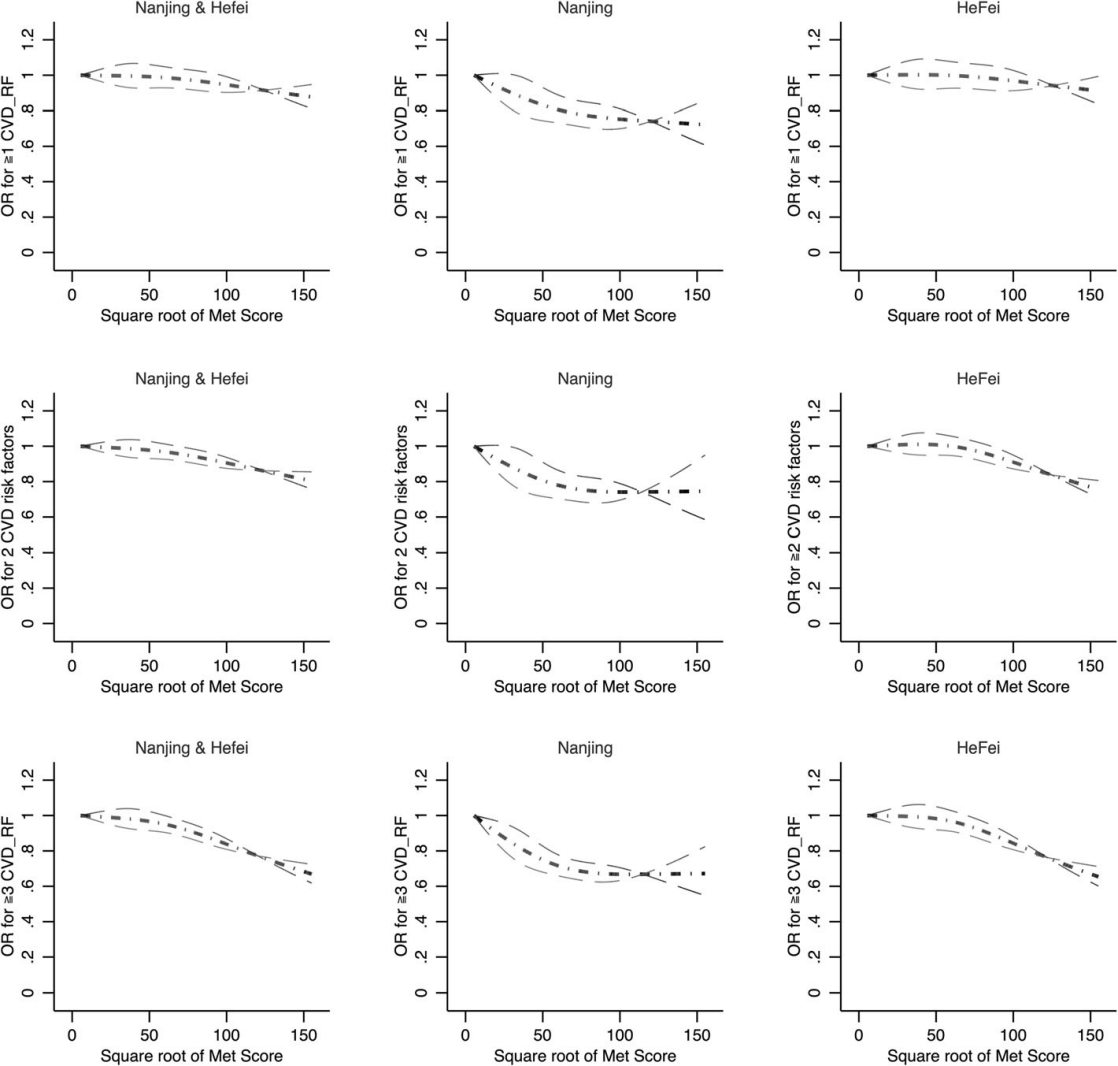
Adjusted dose-response relationship between Met Scores and odds of having ≥1, 2, 3 cardiovascular disease risk factors among Chinese adults. In the pooled dataset, sex, age group and survey site were adjusted; in the survey site specific dataset, sex and age group were adjusted

The dose-response association between transformed Met Score and adjusted odds ratio (OR) of having ≥1, ≥2 and ≥ 3 CVD modifiable risk factors was presented in Fig. 1. Linear dose-response relationships were found in overall, by survey, sex, and age groups (non-linear test: all *P*-value> 0.05).

As presented in Table 3, the adjusted OR of having ≥1, ≥2 and ≥ 3 CVD modifiable risk factors by per SD increase was 0.98 (0.94 to 1.01), 0.96 (0.93 to 0.99) and 0.93 (0.90 to 0.96), respectively. In the site-specific analysis, the significant adjusted OR of having ≥1, ≥2, and ≥ 3 CVD modifiable risk factors was found in Nanjing survey, Hefei survey and both surveys. In the sex-specific analysis, adjusted OR of having ≥1 CVD modifiable risk factors was insignificant in both gender; the adjusted OR of having ≥2, and ≥ 3 CVD modifiable risk factors were both significant in both genders. In the age-stratified analysis, the significant adjusted OR of having ≥1 CVD modifiable risk factors was found in those aged 35–44 years; the adjusted OR of having ≥2, and ≥ 3 CVD modifiable risk factors was significant in those aged 35–44 years and those aged 45–54 years, respectively.

**Table 3:** Adjusted association between physical activity scores (per SD increase) and odds of having ≥1, 2, 3 CVD modifiable risk factor among Chinese adults

	Adjusted Odds ratio (95 confidence interval)		
	Having ≥1 CVD modifiable risk factor	Having ≥2 CVD modifiable risk factors	Having ≥3 CVD modifiable risk factors
Overall ^a^	0.98 (0.94 to 1.01)	0.96 (0.93 to 0.99)	0.93 (0.90 to 0.96)
Survey site ^b^			
Nanjing	0.92 (0.86 to 0.99)	0.95 (0.90 to 1.01)	0.90 (0.83 to 0.96)
Hefei	0.98 (0.94 to 1.03)	0.96 (0.93 to 0.99)	0.93 (0.89 to 0.97)
Sex ^c^			
Male	0.94 (0.87 to 1.01)	0.93 (0.89 to 0.97)	0.90 (0.86 to 0.94)
Female	0.98 (0.93 to 1.03)	0.96 (0.93 to 0.99)	0.94 (0.90 to 0.99)
Age group ^d^			
< 35 years	0.99 (0.97 to 1.02)	0.92 (0.88 to 0.96)	0.72 (0.33 to 1.57)
35–44 years	0.97 (0.95 to 0.99)	0.97 (0.95 to 0.99)	0.93 (0.87 to 0.99)
45–54 years	1.00 (0.97 to 1.03)	0.96 (0.94 to 0.98)	0.92 (0.87 to 0.98)
55–64 years	0.95 (0.90 to 1.00)	0.98 (0.96 to 1.00)	0.97 (0.91 to 1.03)
65–74 years	0.95 (0.89 to 1.01)	0.99 (0.98 to 1.00)	0.95 (0.85 to 1.06)
≥ 75 years	0.68 (0.33 to 1.03)	0.89 (0.75 to 1.03)	0.54 (0.07 to 1.02)

^a^ indicates sex, survey site, and age group were adjusted; ^b^ indicates sex and age group were adjusted; ^c^ indicates survey site and age group were adjusted; ^d^ indicates sex and survey site were adjusted

The odds ratio was estimated by per SD (28 transformed Met Score) increase

In Table 4, the adjusted OR of having ≥1, ≥2, and ≥ 3 CVD modifiable risk factors for moderate physical activity group and high physical activity group was 0.88 (0.79 to 0.98) and 0.88 (0.79 to 0.99), 0.85 (0.78 to 0.92) and 0.85 (0.78 to 0.92), 0.84 (0.76 to 0.91) and 0.81 (0.74 to 0.89), respectively, with low physical activity as reference group. In the stratified analysis, the significant adjusted OR of having ≥2 and ≥ 3 CVD modifiable risk factors was found for moderate physical activity group and high physical activity group in each survey sample, and 35–44 and 45–54 age groups, respectively.

**Table 4:** Adjusted association between physical activity scores category and odds of having ≥1, 2, 3 CVD modifiable risk factors among Chinese adults

	Adjusted Odds Ratio (95% confidence interval)					
	Having ≥1 CVD modifiable risk factor		Having ≥2 CVD modifiable risk factors		Having ≥3 CVD modifiable risk factors	
	Moderate physical activity	High physical activity	Moderate physical activity	High physical activity	Moderate physical activity	High physical activity
Overall ^a^	0.88 (0.79 to 0.98)	0.88 (0.79 to 0.99)	0.85 (0.78 to 0.92)	0.85 (0.78 to 0.92)	0.84 (0.76 to 0.91)	0.81 (0.74 to 0.89)
Survey site ^b^						
Nanjing	0.83 (0.68 to 1.01)	0.79 (0.64 to 0.97)	0.75 (0.65 to 0.88)	0.71 (0.61 to 0.83)	0.71 (0.60 to 0.84)	0.65 (0.55 to 0.78)
Hefei	0.88 (0.77 to 1.01)	0.89 (0.78 to 1.01)	0.87 (0.79 to 0.96)	0.88 (0.80 to 0.96)	0.87 (0.78 to 0.96)	0.84 (0.76 to 0.93)
Sex ^c^						
Male	0.86 (0.78 to 0.96)	0.88 (0.73 to 1.03)	0.83 (0.63 to 1.02)	0.81 (0.72 to 0.91)	0.82 (0.60 to 1.04)	0.80 (0.73 to 0.87)
Female	0.99 (0.87 to 1.13)	0.97 (0.85 to 1.11)	0.97 (0.87 to 1.09)	0.98 (0.87 to 1.09)	0.93 (0.81 to 1.06)	0.95 (0.83 to 1.09)
Age group ^d^						
< 35 years	0.97 (0.30 to 3.09)	0.67 (0.19 to 2.30)	0.27 (0.08 to 0.84)	0.51 (0.16 to 1.61)	0.26 (0.06 to 1.12)	0.33 (0.08 to 1.41)
35–44 years	0.77 (0.65 to 0.90)	0.81 (0.69 to 0.95)	0.69 (0.60 to 0.79)	0.70 (0.61 to 0.80)	0.71 (0.59 to 0.85)	0.69 (0.57 to 0.82)
45–54 years	0.76 (0.62 to 0.95)	0.77 (0.62 to 0.95)	0.77 (0.67 to 0.89)	0.82 (0.71 to 0.95)	0.76 (0.65 to 0.88)	0.69 (0.59 to 0.81)
55–64 years	0.92 (0.70 to 1.20)	0.96 (0.74 to 1.26)	0.98 (0.83 to 1.16)	0.96 (0.81 to 1.13)	0.87 (0.74 to 1.03)	0.90 (0.76 to 1.06)
65–74 years	0.65 (0.41 to 1.03)	0.84 (0.54 to 1.32)	0.98 (0.76 to 1.28)	0.97 (0.75 to 1.27)	1.00 (0.78 to 1.29)	0.91 (0.70 to 1.17)
≥ 75 years	0.54 (0.09 to 3.62)	0.48 (0.09 to 2.54)	0.71 (0.21 to 2.38)	0.83 (0.25 to 2.76)	0.75 (0.22 to 2.56)	0.23 (0.06 to 0.95)

^a^ indicates sex, survey site, and age group were adjusted; ^b^ indicates sex and age group were adjusted; ^c^ indicates survey site and age group were adjusted; ^d^ indicates sex and survey site were adjusted

Adjusted odds ratio was estimated with low physical activity as reference group

## Discussion

Based on two independent surveys from two provinces in China, this study comprehensively examined the dose response associations of self-reported total physical activity and clustering of CVD modifiable risk factors among Chinese adults. Higher prevalence of clustering across CVD modifiable risk factors is associated with lower physical activity. The increase of the physical activity from < 600 MET-min/week to 600–3000 MET-min/week is associated with 12, 15 and 16% decreased odds of having ≥1, ≥2 and ≥ 3 CVD modifiable risk factors. Although prevalence of each CVD modifiable risk factors was different over two surveys, the consistently similar findings are revealed in each survey. Improving physical activity among younger persons before the impacts of chronic disease become largely irreversible, could help to improve life expectancy.

Physical activity and single CVD modifiable risk factors in Chinese population have been addressed in prior studies. For example, Lao et al. identified inverse dose-response association between leisure-time physical activity and diabetes risk as higher levels of leisure-time physical activity are associated with a lower risk of diabetes in Chinese people with impaired fasting glucose [16]. Yang et al. found that household physical activity was inversely associated with the risk of type 2 diabetes in among urban males in northern China [17]. Pang et al. reveals that higher physical activity is associated with lower concentration of atherogenic lipoprotein and cholesterol and lower levels of inflammation in Chinese population [18]. Li et al. found higher level physical activity is related to between glycemic control and insulin sensitivity in southern Chinese population [19]. Similar with these findings, we identified inverse linear dose-response relationship between higher total physical activity level and lower prevalence of having ≥1, ≥2 and ≥ 3 CVD modifiable risk factors among Chinese adults. Instead of measuring parts of daily physical activity (i.e. leisure-time physical activity, sedentary time, or household physical activity), the overall activity measurement incorporating all parts of daily physical activity were undertaken in this study. And instead of investigating the single CVD modifiable risk factors, this study focused on the clustering status of CVD modifiable risk factors, which would generally reflect the individual CVD health and potential future CVD risk. In this study, about 15% decreased prevalence of having ≥2 CVD risk factors might be avoided if the inactive individuals ((< 600 MET-min/week) improved their physical activity to moderate level (600–3000 MET-min/week).

In this study, we investigated the dose-response association between physical activity and clustering of CVD modifiable risk factors not only in the general population but also in the different age-stratified subpopulation. The significant inverse association (higher prevalence of clustering across CVD modifiable risk factors is associated with lower physical activity) was identified in people aged 35–44 years. Among people aged less than 35 years, the insignificant association might suggest that physical activity might not take a significant role in the early onset of clustering of CVD modifiable risk factors [20], which of course need further validation in the external young populations. Among people aged more than 55 years, the existing comorbidities might take a significant role of the clustering of CVD modifiable risk factors [21], which would attenuate the contribution of physical activity. Based on this study, the health promotion programs including improving general physical activities should be advocated in the Chinese population aged 35–54 by health policy makers.

Potential biological mechanism underlying the cardiovascular protective effects of physical activity against the clustering of CVD modifiable risk factors include some favorable modification effects in adiposity, insulin sensitivity, lipid profiles and systemic inflammation [16, 22–24]. Experimental studies also revealed that physical activity helps to improve body muscle mass and stimulates the uptake of glucose in muscles [25, 26] and reduce triglycerides and ceramides and improve insulin sensitivity [27].

As the strength of this study, overall > 90% response rate was achieved by this study, which made the study sample well represent local populations. The high response rate was achieved by household investigation and face-to-face interview by trained research staff. The limitation of this study is the utilization of cross-sectional survey datasets, whereby physical activity measurements and CVD modifiable risk factors were evaluated at same time. Therefore, it is impossible to process causal inference between physical activity and clustering of CVD modifiable risk factors. Future studies within in prospective cohort data would be the next step in testing above associations. Some other confounders like comorbidities (like existing CVD) could also alter the association between physical activity and clustering of CVD modifiable risk factors. However, more comorbidities more not available in this study. Finally, future external replication studies with adjustment of potential comorbidities, alongside meta-analysis are warranted to better understanding whether physical activity does have the potential modification role in the clustering of CVD modifiable risk factors. Some other blood markers that could confound the findings of this study, like C-reactive protein were not accessible in this study and its confounding effect could not be addressed in this study. Instead of using International Physical Activity Questionnaire to assess physical activity, accelerometers or other wearables might be preferred in recent studies. Future replication studies using wearables to assess physical activity are warranted.

## Conclusion

In conclusion, in Chinese adults, physical activity level inversely associates with clustering of CVD modifiable risk factors, especially in Chinese adults aged 35 to 54 years. Health promotion programme including improve physical activity level should be advocated. Physical activity has the potential modification role in the clustering of CVD modifiable risk factors that warrants further validation in the future prospective cohort studies.

## Data Availability

The data used and/or analyzed during the study are available from the corresponding author on reasonable request.

## Abbreviations

CVD: Cardiovascular diseases
CVDMRF: Clustering of cardiovascular diseases modifiable risk factors
MET: Metabolic equivalent task
BMI: Body mass index
SBP: Systolic blood pressure
DBP: Diastolic blood pressure
OR: Odds ratio
CI: Confidence interval
